# A Linked Population-Based Analysis of Severe Lower Extremity Traumas in Ontario

**DOI:** 10.1177/22925503231217515

**Published:** 2023-11-30

**Authors:** Stephanie M. Kim, Andrew McClure, Jennifer Reid, Luc Dubois, Abdel-Rahman Lawendy, Andrew M. Simpson

**Affiliations:** 1 Division of Plastic and Reconstructive Surgery, Department of Surgery, 6221 Western University, London, ON, Canada; 2 Department of Surgery, 10033 London Health Sciences Centre, London, ON, Canada; 3 ICES Western, 6221 Western University, London, ON, Canada; 4 Division of Vascular Surgery, Department of Surgery, 6221 Western University, London, ON, Canada; 5 Division of Orthopaedic Surgery, Department of Surgery, 6221 Western University, London, ON, Canada

**Keywords:** open tibia fracture, extremity fracture, soft tissue reconstruction, orthoplastic surgery, fracture ouverte du tibia, fracture d’un membre, reconstruction des tissus mous, chirurgie orthoplastique

## Abstract

**Background:** Successful management of open tibial fractures often requires a multidisciplinary approach. Previous literature has shown that admission to a trauma centre and coordinated management of early bony fixation and soft-tissue reconstruction improve patient outcomes. The objective of this study was to describe patients who had open tibial fractures in Ontario and analyse their outcomes. **Method:** Linked population-based administrative data pertaining to patients who had open tibia fractures between the years 2009 and 2020 were examined. Demographic information, admission location, timing of soft-tissue reconstruction, and complication rates were collected. **Results:** 4240 patients were identified. The mean age was 47, and the majority were males. One-third of the patients were admitted to a trauma centre. This group were more likely to have an Injury Severity Score >15, and associated neurovascular injuries of the leg. When outcomes were compared over 1 year, the trauma centre group was more likely to undergo limb amputation, but also more likely to have a soft-tissue reconstruction. The average time to soft-tissue reconstruction was 20 days. Patients receiving care in a trauma centre were more likely to have an external fixation, plastic surgery consults, and longer hospital stay. There were no differences in the odds of infection, malunion or nonunion, and 30-day mortality. **Conclusions:** These findings provide a comprehensive examination of the clinical course for patients experiencing open tibia fractures in Ontario. Future studies can help facilitate coordinated efforts between different specialties to expedite care and improve overall outcomes.

## Résumé

**Contexte**: une gestion réussie des fractures ouvertes du tibia nécessite souvent une approche multidisciplinaire. Les publications antérieures ont montré que l’hospitalisation dans un centre de traumatologie et une gestion coordonnée pour une fixation osseuse précoce et une reconstruction des tissus mous améliorent les résultats des patients. L’objectif de cette étude était de décrire des patients victimes de fractures ouvertes du tibia en Ontario et d’analyser leurs résultats. **Méthode**: Les données administratives liées basées sur la population et liées concernant des patients qui avaient eu des fractures ouvertes du tibia entre 2009 et 2020 ont été examinées. Les renseignements démographiques, l’emplacement de l’hospitalisation, le moment de la reconstruction des tissus mous et les taux de complications ont été collectés. **Résultats**: 4240 patients ont été identifiés. L’âge moyen était de 47 ans, en majorité de sexe masculin. Un tiers des patients a été admis dans un centre de traumatologie. Ce groupe était plus susceptible d’avoir une cote de sévérité de la blessure > 15 avec des lésions neurovasculaires associées de la jambe. Quand les résultats ont été comparés sur 1 an, le groupe du centre de traumatologie a été plus susceptible de subir une amputation de membre et aussi plus susceptible de bénéficier d’une reconstruction des tissus mous. Le délai moyen pour la reconstruction des tissus mous était de 20 jours. Les patients recevant des soins dans un centre de traumatologie ont été plus susceptibles d’avoir une fixation externe, une consultation en chirurgie plastique et une plus longue durée d’hospitalisation. Il n’y a pas eu de différences pour les risques d’infection, de pseudarthrose ou de non-soudure osseuse et de mortalité à 30 jours. **Conclusions**: Ces constatations procurent un examen complet de l’évolution clinique des patients victimes de fractures ouvertes du tibia en Ontario. Des études futures pourront contribuer à faciliter les efforts combinés entre différentes spécialités pour accélérer les soins et améliorer les résultats globaux.

## Introduction

Open tibial fractures refer to tibia fractures associated with soft-tissue injuries. These fractures can be difficult to manage, often requiring a collaborative effort between orthopedic surgery and plastic surgery teams for early irrigation, debridement, bony stabilization, and soft-tissue reconstruction. Soft-tissue reconstruction can range from local and regional flaps to more complicated free flaps, and is essential for restoring vascularity, stability, and providing coverage for vital structures such as bone, nerves, and vessels. These surgeries are typically done by plastic and reconstructive surgeons and completed after bony fixation by orthopedic surgeons. Due to the closely integrated and collaborative relationship between the two specialties, many institutions in the United Kingdom and other parts of the world have adopted “orthoplastic” teams to help manage these injuries in more efficient and streamlined manner.^1-3^

Despite multidisciplinary efforts, open tibial injuries confer high complication rates including infections, bony malunion or nonunion, compartment syndrome, need for revision surgery and amputation. The complexity of management and the high rates of complications have prompted vast research into coordination and centralization of care to examine and improve outcomes in open tibial fractures. Early surgical debridement, direct admission to a trauma centre with appropriate specialists available, and early soft-tissue reconstruction have been shown to reduce complication rates, number of surgeries, and length of hospital stay.^4-7^

A number of population-level studies have examined open tibial fracture management in the United States and Europe,^8-13^ but similar studies have not been performed in Canada. The characteristics of the patient population and typical management patterns in Ontario remain largely unknown. The goal of this work is to describe the characteristics of individuals with open tibial injuries in Ontario, analysing their admission locations, management patterns, complication rates, and investigating areas where care could be improved.

## Methods

To conduct this study, data from ICES, previously known as the Institute for Clinical Evaluative Sciences, was used. ICES is a nonprofit research institute that holds administrative population-based linked databases, retaining a variety of health-related data for every single individual in Ontario. These datasets were linked using unique encoded identifiers and analysed at ICES. These include demographic information, different hospital encounters, diagnoses, and interventions the individual had received. For our study, those who sustained an open tibial fracture between April 1, 2009 to March 31, 2020 were identified using ICD-10 diagnostic codes (S82101, S82201, S82301). Patients were excluded if the data was incomplete, they were not Ontario resident, were aged less than 18 or greater than 105, or had previous tibia injury. Patients who did not have a record of visit to the emergency department, or a record in the Ontario Trauma Registry, as well as the record of admission to the hospital were excluded. Other than records from individual hospital emergency department, Ontario Trauma Registry collaterally records administrative data related to major traumas in Ontario and was cross-reviewed to ensure no pertinent information was missed.

Basic demographic information, relevant past medical history, and details about the injury were examined. Year of injury, mechanism of injury, presence/type of neurovascular injury, and whether the Injury Severity Score (ISS) was greater than 15 were collected. To investigate what type of hospitals they were being admitted to, we examined those admitted to trauma centre, and nontrauma centres. Nine adult trauma centres in Ontario (Winsor Regional Hospital, London Health Sciences Center, Hamilton Health Sciences, St. Michael's Hospital, Sunnybrook Health Sciences Center, Kingston General Hospital, The Ottawa Hospital, Health Sciences North, and Thunder Bay Regional Health Sciences Center) were included in the analysis.^14^ One of the major outcomes that was examined was whether one had a soft-tissue reconstruction in the hospital, and if it occurred in the timeframe of 72 h. In addition, the incidence of amputation, malunion or nonunion, debridement for infection, compartment syndrome, external fixator placement, death, consultation with plastic surgery service, and length of stay in the hospital were examined.

Baseline variables were compared between those admitted to a trauma centre and a nontrauma centre using one-way analysis of variance, Kruskal–Wallis, chi-square, and the Cochran-Armitage trend test. For outcomes, Mann-Whitney *U* analysis was completed for continuous variables, and the odds ratio (OR) was calculated for the categorical variables. Standardized differences were also reported as a subtle measure of differences in the results where the sample size was large.^15^ Regression models were further used to analyse the outcome of the two groups, those admitted to a trauma centre and a nontrauma centre. Covariates such as direct admission to a trauma centre, patient age, sex, Charlson Comorbidity Index (CCI), rurality, ISS > 15, neurovascular injury, and mechanism of injury were included.

## Results

A total of 4240 patients who had open tibial fractures and met the inclusion and exclusion criteria were identified. When stratified by admission location, 1722 patients were admitted to a trauma centre, and 2471 patients were admitted to a nontrauma centre. The baseline characteristics of the cohort population are described in Table 1. The mean age of the cohort population was 47, and more than two-thirds of the patients were males. Motor-vehicle collisions (MVC) were the most common cause of the injury, accounting for almost half of the identified patients, followed by falls.

**Table 1.** Baseline Variables of the Cohort Population.

Variable	Value	Non-TC	TC	Transfer to TC	Overall	TC versus non-TC	
		*N* = 2471	*N* = 1722	*N* = 47	*N* = 4240	St.Di	*P* value
Patient age	Mean (SD)	48.28 ± 18.87	45.63 ± 18.08	49.55 ± 18.03	47.22 ± 18.59	0.14	<.001
	Median (IQR)	48 (32-62)	45 (30-58)	47 (38-64)	47 (31-60)	0.14	<.001
Patient sex	Female	842 (34.1%)	476 (27.6%)	21 (44.7%)	1339 (31.6%)	0.14	<.001
	Male	1629 (65.9%)	1246 (72.4%)	26 (55.3%)	2901 (68.4%)	0.14	
Rural resident	Yes	412 (16.7%)	309 (17.9%)	13 (27.7%)	734 (17.3%)	0.03	.353
Income quintile	Missing	11 (0.4%)	12 (0.7%)	0 (0.0%)	23 (0.5%)	0.03	.004
	Quintile 1	518 (21.0%)	441 (25.6%)	6 (12.8%)	965 (22.8%)	0.11	
	Quintile 2	503 (20.4%)	365 (21.2%)	8 (17.0%)	876 (20.7%)	0.02	
	Quintile 3	513 (20.8%)	317 (18.4%)	≤20	≤850	0.06	
	Quintile 4	500 (20.2%)	309 (17.9%)	≤5	≤820	0.06	
	Quintile 5	426 (17.2%)	278 (16.1%)	13 (27.7%)	717 (16.9%)	0.03	
Charlson commor-bidity index	Mean (SD)	0.17 ± 0.72	0.15 ± 0.70	0.09 ± 0.41	0.16 ± 0.71	0.02	.563
	Median (IQR)	0 (0-0)	0 (0-0)	0 (0-0)	0 (0-0)	0.02	.529
	0	2288 (92.6%)	1603 (93.1%)	≤5	≤3940	0.02	.657
	1	77 (3.1%)	56 (3.3%)	0 (0.0%)	133 (3.1%)	0.01	
	2	55 (2.2%)	29 (1.7%)	≤5	≤90	0.04	
	3+	51 (2.1%)	34 (2.0%)	0 (0.0%)	85 (2.0%)	0.01	
History of hypertension	Yes	693 (28.0%)	406 (23.6%)	9 (19.1%)	1108 (26.1%)	0.1	.001
History of diabetes	Yes	303 (12.3%)	192 (11.1%)	≤5	≤5	0.03	.272
History of CHF	Yes	104 (4.2%)	63 (3.7%)	0 (0.0%)	167 (3.9%)	0.03	.37
History of COPD	Yes	278 (11.3%)	168 (9.8%)	≤5	≤455	0.05	.123
History of peripheral vascular disease	Yes	13 (0.5%)	13 (0.8%)	0 (0.0%)	26 (0.6%)	0.03	.353
Injury severity score >15	Yes	365 (14.8%)	1010 (58.7%)	9 (19.1%)	1384 (32.6%)	1.02	<.001
Associated neurovascular injury	Yes	32 (1.3%)	98 (5.7%)	≤5	≤135	0.24	<.001
Nerve injury	Yes	11 (0.4%)	18 (1.0%)	≤5	≤35	0.07	.021
Vascular injury	Yes	24 (1.0%)	89 (5.2%)	≤5	≤115	0.25	<.001
Mechanism of injury	MVC	803 (32.5%)	1078 (62.6%)	25 (53.2%)	1906 (45.0%)	0.63	<.001
	Fall	1106 (44.8%)	307 (17.8%)	16 (34.0%)	1429 (33.7%)	0.61	
	Others	562 (22.7%)	337 (19.6%)	6 (12.8%)	905 (21.3%)	0.08	
Fiscal year	2009	232 (9.4%)	171 (9.9%)	8 (17.0%)	411 (9.7%)	0.02	.033
	2010	219 (8.9%)	151 (8.8%)	6 (12.8%)	376 (8.9%)	0	
	2011	235 (9.5%)	141 (8.2%)	8 (17.0%)	384 (9.1%)	0.05	
	2012	214 (8.7%)	140 (8.1%)	≤5	≤360	0.02	
	2013	235 (9.5%)	131 (7.6%)	9 (19.1%)	375 (8.8%)	0.07	
	2014	213 (8.6%)	141 (8.2%)	≤5	≤360	0.02	
	2015	213 (8.6%)	170 (9.9%)	≤5	≤390	0.04	
	2016	202 (8.2%)	179 (10.4%)	≤5	≤385	0.08	
	2017	204 (8.3%)	173 (10.0%)	≤5	≤380	0.06	
	2018	266 (10.8%)	163 (9.5%)	0 (0.0%)	429 (10.1%)	0.04	
	2019	238 (9.6%)	162 (9.4%)	≤5	≤405	0.01	

Abbreviations: MVC, motor-vehicle collision; TC, trauma centre.

When the characteristics were compared between those who were admitted to trauma centre versus nontrauma centre, the trauma centre group had a lower mean age, greater proportion of males, and more likely to have been admitted from MVC. The trauma centre group also had a greater rate of associated neurovascular injury.

Different management patterns such as whether the individual had a soft-tissue reconstruction, external fixation, or amputation were assessed. Their long-term outcomes, such as incidence of infections, malunion or nonunion, and compartment syndrome for up to 1 year were also assessed. Table 2 shows the unadjusted odds of the above outcomes between those directly admitted to TC compared to those admitted to nontrauma centre. Our analysis demonstrated that greater proportion of the trauma centre group had soft-tissue reconstruction in 90 days (OR 3.54, *P* < .0001). The trauma centre group also had a greater proportion of people who had very early soft-tissue coverage within 72 h (OR 1.75, *P* = .0066). In contrast to the total proportion being larger, the trauma centre group had longer time to soft-tissue surgery, compared to those who had surgery in a nontrauma centre. The trauma centre group also had a longer stay in the hospital as demonstrated in Table 3.

**Table 2.** Categorical Outcomes of Individuals with Open Tibia Fractures, Comparing Those who Were Admitted to a TC to Those Admitted to a non-TC.

Outcome	Overall	Non-TC	TC	Transfer to a TC	TC to reference (non-TC)	
					OR (95% CI)	*P*-value
Soft tissue reconstructive surgery	416 (9.8%)	127 (5.1%)	277 (16.1%)	12 (25.5%)	3.54 (2.84-4.41)	<.0001
Soft tissue reconstructive surgery within 72 h	97 (2.3%)	44 (1.8%)	53 (3.1%)	0	1.75 (1.17-2.63)	.0066
Amputation within 7-days	118 (2.8%)	22 (0.9%)	96 (5.6%)	0	6.57 (4.12-10.45)	<.0001
Amputation within 1-year	192 (4.5%)	54 (2.2%)	138 (8.0%)	0	3.90 (2.83-5.37)	<.0001
Wound infection/debridement	1433 (33.8%)	738 (29.9%)	669 (38.9%)	26 (55.3%)	1.49 (1.31-1.70)	<.0001
Diagnosis of malunion or nonunion	356 (8.4%)	190 (7.7%)	158 (9.2%)	8 (17.0%)	1.21 (0.97-1.51)	.0865
Compartment syndrome	≤265	136 (5.5%)	122 (7.1%)	≤5	1.31 (1.02-1.69)	.0365
External fixator placement	766 (18.1%)	282 (11.4%)	461 (26.8%)	23 (48.9%)	2.84 (2.41-3.34)	<.0001
Death within 30-days	111 (2.6%)	36 (1.5%)	75 (4.4%)	0	3.08 (2.06-4.61)	<.0001
Consultation with plastic surgery service	880 (20.8%)	309 (12.5%)	552 (32.1%)	19 (40.4%)	3.30 (2.82-3.86)	<.0001

Some exact numbers are not reported as per ICES policy to ensure confidentiality. Abbreviations: OR, odds ratio; TC, trauma centre; CI, confidence interval.

**Table 3.** Continuous Outcomes

Outcome	Overall	Non-TC	TC	Transfer to a TC	*P*-value
	Mean (SD)	Mean (SD)	Mean (SD)	Mean (SD)	
Length of hospital stay (days)	14.0 (20.7)	9.7 (13.5)	20.4 (27.0)	5.7 (3.5)	<.0001
Time to soft-tissue reconstruction (days)	19.5 (21.1)	17.4 (21.5)	20.6 (21.2)	17.4 (9.1)	.0073

Regression analysis was performed considering multiple variables that were found significant in the initial baseline analysis. They were used to identify adjusted odds of patient outcomes. When adjusted, there was still significantly higher odds (OR 1.89, *P* < .0001) of having a soft-tissue reconstructive surgery in a trauma centre versus nontrauma centre in 90 days (Table 4). When we examined soft-tissue reconstruction within 72 h, admission location did not have a significant effect. There were increased odds of amputation in a trauma centre, for both early (7 days, OR 2.32) and late time period (1 year, OR 1.79). We saw that in addition to admission location, other associated injuries such as whether ISS was greater than 15 or not, and the presence of a nerve and/or vascular injury affected the odds of amputation (Table 5).

**Table 4.** Regression Analysis on Soft Tissue Reconstruction and Those Within 72 h.

Covariate	Soft tissue reconstruction surgery				Soft tissue reconstruction <72 h			
	OR	95% CI		*P*-value	OR	95% CI		*P*-value
TC admission (yes vs no)	1.89	1.47	2.42	<.0001	0.90	0.57	1.43	.6556
Age	1.00	0.99	1.01	0.7499	1.00	0.99	1.01	.9253
Sex (male vs female)	1.42	1.09	1.86	0.0091	1.45	0.87	2.41	.1594
Rural residency (vs urban)	1.08	0.82	1.42	0.588	1.06	0.63	1.79	.8272
ISS > 15 (yes vs no)	2.18	1.67	2.83	<.0001	2.31	1.39	3.86	.0013
CCI	0.95	0.78	1.15	0.5935	0.78	0.46	1.34	.3747
Neurovascular injury (yes vs no)	3.51	2.38	5.16	<.0001	2.74	1.42	5.30	.0027
Mechanism of injury (fall vs MVC)	0.37	0.25	0.54	<.0001	0.43	0.22	0.87	.018
Mechanism of injury (other vs MVC)	0.96	0.73	1.27	0.7966	0.86	0.50	1.47	.5812

Abbreviations: ISS, Injury Severity Score; CCI, Charlson Comorbidity Index; MVC, motor-vehicle collision.

**Table 5.** Regression Analysis on Amputation as Outcome.

Covariate	Amputation within 7-days				Amputation within 1 year			
	OR	95% CI		*P*-value	OR	95% CI		*P*-value
TC admission (yes vs no)	2.32	1.39	3.86	0.0013	1.79	1.23	2.59	.0022
Age	1.01	1.00	1.02	0.2373	1.01	1.00	1.02	.0039
Sex (male vs female)	1.03	0.66	1.61	0.8922	1.18	0.83	1.69	.3613
Rural residency (vs urban)	1.09	0.67	1.78	0.7306	1.26	0.86	1.85	.2371
ISS > 15 (yes vs no)	5.16	2.88	9.26	<.0001	3.60	2.37	5.46	<.0001
CCI	1.09	0.82	1.44	0.5693	1.33	1.15	1.54	.0002
Neurovascular injury (yes vs no)	4.76	2.90	7.83	<.0001	7.61	4.96	11.68	<.0001
Mechanism of injury (fall vs MVC)	0.26	0.10	0.69	0.0064	0.77	0.47	1.25	.2898
Mechanism of injury (other vs MVC)	1.14	0.70	1.88	0.5979	0.96	0.62	1.47	.8395

Abbreviations: ISS, Injury Severity Score; CCI, Charlson Comorbidity Index; MVC, motor-vehicle collision.

Other complication rates examined are summarized in Table 6. Admission location did not have a significant impact on the odds of infection requiring debridement or hardware removal. Admission location also did not influence the odds of malunion or nonunion. In contrast, we saw trauma centre admission significantly increased odds of having external fixation. and greater odds of having a plastic surgery consultation. Compartment syndrome was the only complication that had decreased odds in the trauma centre group. Overall, other covariates such as having ISS > 15 and the presence of neuro-vascular injury had much greater impact on the odds of developing major complications including wound infection and compartment syndrome, and placement of external fixator.

**Table 6.** Regression Analysis on Other Secondary Variables Including Wound Infection, External Fixation, Plastic Surgery Consultation, Compartment Syndrome, Malunion or Nonunion, and Death Within 30 Days.

Covariates	Odds ratios of outcomes					
	Wound infection/debride-ment	External fixation	Plastic surgery consult	Compartment syndrome	Malunion and nonunion	Death within 30 days
TC admission (yes vs no)	1.02	1.72*	1.64*	0.55*	1.04	1.58
Age	1.00	1.00	1.00	0.99	1.00	1.04*
Sex (male vs female)	1.21*	1.03	1.29*	1.68*	1.50*	0.71
Rural residency (vs urban)	1.32*	1.69*	0.95	0.93	1.16	1.28
ISS > 15 (yes vs no)	1.84*	3.15*	2.62*	3.07*	1.11	6.83*
CCI	1.05	0.97	0.96	1.16	0.90	1.42*
Neurovascular injury (yes vs no)	2.36*	2.14*	3.82*	6.04*	1.52	0.56
Mechanism (fall vs MVC)	0.84	1.15	0.38*	0.45*	0.83	1.03
Mechanism (other vs MVC)	0.88	0.82	0.64*	1.17	0.88	0.36*

*Notes significant ORs with *P*-value <.05. Abbreviations: ISS, Injury Severity Score; CCI, Charlson Comorbidity Index; MVC, motor-vehicle collision.

30-day mortality was assessed in this population. While trauma centre admission did not result in significant differences in the odds of death within 30 days, ISS > 15, age, and pre-existing comorbidities as represented by CCI, were significantly associated with increased odds (Table 6). Lastly, the length of stay in the hospital was examined. Due to the significant overdispersion of the variable, negative binomial model was used instead of a regression model. While all variables affected the length of hospital stay, trauma centre admission was associated with increased length of stay (Table 7).

**Table 7.** Binomial Regression Analysis on Length of Stay in the Hospital.

Covariate	Length of hospital stay	
	Estimate	*P*-value
TC admission (yes vs no)	0.40	<.0001
Age	0.01	<.0001
Sex (male vs female)	−0.10	.0004
Rural residency (vs urban)	−0.16	<.0001
ISS > 15 (yes vs no)	0.78	<.0001
CCI	0.13	<.0001
Neurovascular injury (Yes vs No)	0.37	<.0001
Mechanism of injury (Fall vs MVC)	−0.14	<.0001
Mechanism of injury (Other vs MVC)	−0.22	<.0001

Abbreviations: ISS, Injury Severity Score; CCI, Charlson Comorbidity Index; MVC, motor-vehicle collision.

## Discussion

Open extremity injuries are challenging to manage. Open tibial fractures are known for high rates of complications including infection, osteomyelitis and amputation.^16-19^ Expedited management with a coordinated approach by multiple experienced personnel is integral for the best patient outcome. This work aimed to describe the patient population affected by these injuries in Ontario describe their management pathways including the admission location, soft-tissue reconstruction, and rates of complications.

Utilizing the ICES, a linked-population level database, we identified our cohort population in Ontario, with all health data gathered for one year following injury. In addition to demographics and injury characteristics, the role of soft-tissue reconstruction was examined. We saw that 9.8% of those with open tibial fractures received reconstructive surgery for their soft tissue injuries. This is similar to a nationwide study in Sweden where 9% of open lower extremity injuries had significant soft tissue injury to require soft-tissue reconstruction.^12^

Early soft-tissue reconstruction in open extremity injuries has been shown to improve patient outcomes.^12,17,20-28^ In our cohort of patients who had soft-tissue reconstruction, 97 out of 416 (23%) had the surgery within 72 h. Overall, the mean time to the surgery was 19.5 days for the entire cohort. While long, this was consistent with recent literature that time to definitive reconstructive surgery can range up to 90 days, especially with the recent use of negative pressure wound therapy.^28-32^ Ontario patients in this cohort undergoing management at a trauma centre had increased time from injury to soft-tissue reconstruction, which was in contrast to previous literature that showed tertiary centres with both orthopedic and plastic surgery specialists contribute to earlier definitive management.^2,3^ Prolonged duration between injury and definitive soft-tissue reconstruction may be from a number of factors, including critical illness, human or infrastructure availability, or local care patterns.^33,34^

Amputation after open tibial fractures is common. In our analysis, we looked at a 7-day timeframe to capture primary amputation, which we defined as amputation without an attempt at limb salvage. We saw significantly increased odds of primary amputation in patients managed at trauma centre, likely related to the severity of their injuries as seen in the regression analysis. When total amputation rate was examined, 4.5% of our cohort had amputation in the first 1 year. This is comparable to previous reports in the literature, where the amputation rate of open tibial fractures is between 3.6% and 8%.^5,35-37^ Although the majority of amputations likely occur in the first year following an open tibial fracture, we hypothesize that there will be instances of amputation outside of the timeframe examined as a result of osteomyelitis and chronic wounds. Further research in this aspect of care is warranted.

Wound infection is one of the most common complications of open extremity injuries, with the reported rates as high as 34.3%.^18,19,38,39^ The incidence of wound infection in our cohort was 33.8%. While admission location did not appear to have a significant impact on the likelihood of infection, the severity of injury as indicated by ISS and the presence of neuro-vascular injury significantly increased the odds of requiring irrigation and hardware removal. Complex injuries are often heavily contaminated and require numerous staged operations for debridement, orthopedic fixations, and reconstructions. These increase exposure to infections, which all could have contributed to our finding. Our methodology, which looked at patients who underwent irrigation and debridement or hardware removal, has limitations. It overestimates the infection cases since not all hardware removal is due to infection, and sometimes due to pain or malunion.^40,41^ On the contrary, it may also underestimate, as some infections do not require surgical debridement and are managed medically with antibiotics.

It was previously shown that lower extremity trauma patients who were directly admitted to a trauma centre had a shorter hospital stay.^4,11^ In our analysis, we saw the length of stay was longer in this group. The discrepancy in our results versus the other literature is likely because they compared those directly admitted to a trauma centre, to those who were transferred from a peripheral centre. On the other hand, our study compared those directly admitted to a trauma centre, to those directly admitted to a nontrauma centre. In our regression analysis, all examined co-variates including admission location, age, sex, severity of injury, and mechanism contributed to the length of stay in the hospital. These findings are consistent with factors previously discussed in the literature, with tertiary centre admissions being impacted by more complex injury,^13^ complications, and repeat operations.^4,11,42^ Patient factors, such as increasing age and comorbidities also affect the length of stay, which we saw in our analysis.^43,44^

A general limitation of the study is that we were not able to account for other bodily injuries. While the ISS > 15 premium-fee code was used to adjust for other injuries, more granular information would have been valuable in the analysis. Additionally, we used administrative codes, and discrepancies can exist between what was completed in the hospital and what was recorded.^45^ In addition, some of the procedures for soft-tissue reconstruction are not always well-captured by the simplified procedure codes that get documented. Future studies can address these limitations and improve methodology to better investigate extremity traumas and their outcomes.

Overall, the current study highlights the importance of close, integrated working relationships of orthopedic and plastic surgery teams in managing complex lower extremity traumas. Centralization of complex care to major tertiary centres has been proposed to expedite care, where dedicated specialists are available for assessments and management. Our analysis showed that soft-tissue reconstruction can still be significantly delayed in trauma centres, likely due to a variety of reasons including critical illness, concomitant injuries, and lack of hospital resources such as staffing. While the provision of complex care may be more available in larger centres, without the coordination of teams and resources, delays may occur, unnecessarily prolonging the time to reconstruction and increasing the likelihood of complications.^10,17,27,46^

A new concept that could be entertained to optimize care in this patient population is an “orthoplastic” surgery team. The orthoplastic team, as the name suggests, would have both orthopedic and plastic surgeons that would assess lower extremity traumas simultaneously for the need for bony and soft tissue surgery early in the trauma triage process. In addition to the accelerated assessments, a dedicated operating time where such multidisciplinary team can work concurrently, can ultimately save resources for the healthcare system. This model has been highly successful in other public healthcare settings,^1-3^ and something that could be adopted in Canada in high volume trauma centres.

## Conclusion

This study is the first of its kind to analyse lower extremity trauma management in Ontario. It provides evidence for initiating conversations on trauma triage and management at an institutional and provincial level to discuss new guidelines and policies for best outcomes in extremity traumas. These results may be used as a baseline for future studies, including implementation of interventions to improve outcomes. There remains room for improvement in aspects of open fracture management, including encouraging better coordination between different surgical teams, and facilitating earlier soft-tissue reconstruction for best patient outcomes.
